# А Rare Case of an Infected, Ruptured Popliteal Artery Aneurysm Occurring Following Surgical Treatment for Panaritium

**DOI:** 10.7759/cureus.54798

**Published:** 2024-02-24

**Authors:** Alexander T Daskalov

**Affiliations:** 1 Vascular Surgery, Acibadem City Clinic Tokuda Hospital, Sofia, BGR

**Keywords:** bypass surgery, sepsis, ascending lymphangitis, stent-graft infection, infected popliteal aneurysm

## Abstract

We present a successful case of treating an infected popliteal aneurysm in a 71-year-old man who arrived at the emergency department in a septic state, reporting a three-week history of fever, lethargy, general malaise, and pain and swelling in the right popliteal fossa. Previously diagnosed with a sizable right popliteal aneurysm, the patient had undergone endovascular treatment using a Viabahn (WL Gore & Associates, Flagstaff, USA) endoprosthesis two months earlier. His fever and malaise emerged a week following minor surgery for a toe infection (panaritium) on the right foot, leading to subsequent necrotic lymphangitis on the dorsum of the same foot. A PET/CT scan strongly indicated an infection within the aneurysmal sac, while a CT angiography confirmed the integrity of the stent graft without any leaks but revealed a ruptured aneurysm. Urgent surgical intervention was necessary. An extra-anatomical autovenous bypass was conducted, followed by an aneurysm and endograft removal. Subsequently, a vacuum-assisted closure (VAC) system was employed to manage the infected wound post sac extraction. The surgical procedure went smoothly without complications, and following a course of antibiotics, the patient recovered well, eventually being discharged after 50 days.

## Introduction

An infected popliteal aneurysm is a rare yet high-risk condition that can lead to emergencies, often resulting from acute ruptures and sepsis. Managing a complicated popliteal aneurysm, especially when accompanied by both local and systemic sepsis, poses significant challenges. Controlling the infection and providing appropriate surgical intervention are crucial aspects of patient care [1]. Open surgical procedures and endovascular treatment are well-established methods to address popliteal artery aneurysms [1,2]. However, it is crucial to note that complications from endovascular treatments may include stent occlusion and the development of endoleaks, leading to aneurysm expansion and potential rupture. Stent graft infection is a rare complication, especially when associated with an ascending infection originating from a purulent process in the forefoot [3,4].

## Case presentation

**Figure 1:**
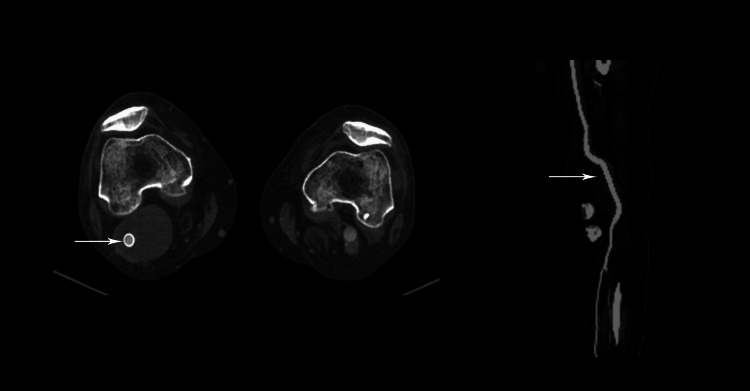
Patent stent graft. No endoleak is present.

A 71-year-old man arrived at the emergency department in a septic state, having experienced fever, lethargy, and overall discomfort for three weeks. He also reported erythema, swelling, and pain in the right popliteal fossa, along with limited knee flexion for the past 10 days. This was his third visit to our department; the initial one occurred seven months ago when he underwent endovascular aortic repair (EVAR) for aortic and bilateral iliac aneurysms. During the initial CT scan, a sizable right popliteal aneurysm measuring 6 by 10 centimeters was detected. Two months ago, this was addressed through endovascular methods using two overlapping endografts: Viabahn 10/100mm distal and 9/100mm proximal (WL Gore & Associates, Flagstaff, AZ, USA). We opted for an endovascular approach considering the patient's history, which includes a prior myocardial infarction followed by coronary revascularization, as well as left ventricular aneurysm resection and an ischemic stroke six months ago. He was discharged without complications, and a follow-up CT scan a month later confirmed the integrity of the stent grafts without any leak (Figure 1).

A month before his current visit, he underwent minor surgery in another hospital to treat a purulent infection (panaritium) in the right toe, necessitating nail extraction. The pus sample tested positive for Serratia marcescens. Subsequently, a week later, he developed painful erythema and swelling on the dorsal surface of the right foot, accompanied by fever and a septic condition. Blood culture tests confirmed the presence of Serratia. Although he received antibiotic treatment (vancomycin IV for about two weeks), his symptoms did not improve.

**Figure 2:**
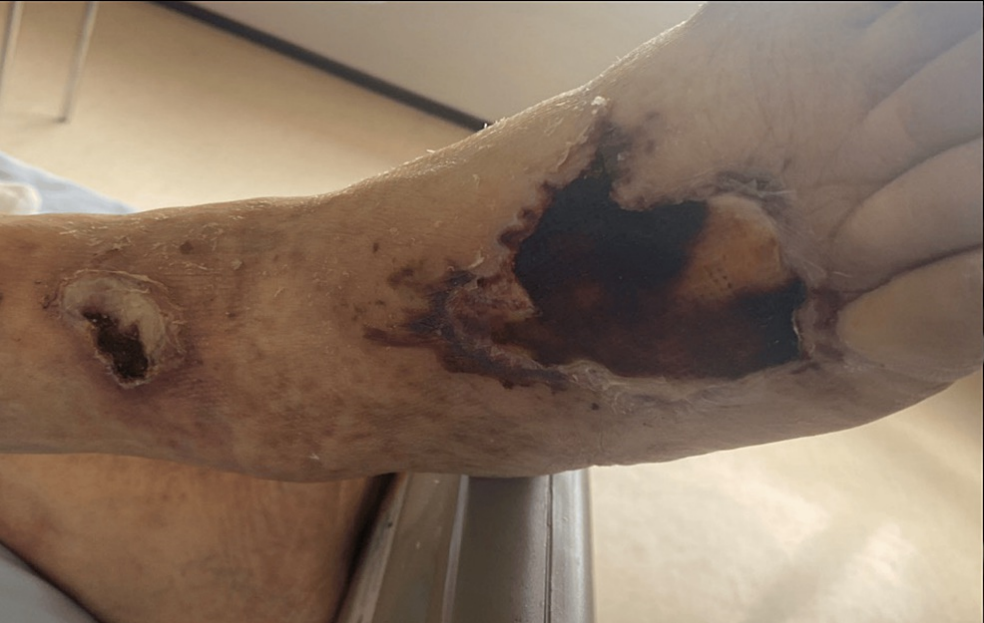
Necrotic lesion on the dorsum of the right foot.

Upon admission to our unit, a clinical examination revealed several concerning findings: the patient exhibited fever, a painful swelling in the left popliteal fossa, and signs indicative of local inflammation. Additionally, evident on the right foot dorsum was a clinical manifestation of lymphangitis complicated by skin necrosis (Figure 2). 

Laboratory analysis demonstrated a notably elevated white blood cell count of 34.89 x 10^9/L and a C-reactive protein level of 73.54 mg/L. The patient's preoperative ankle-brachial pressure index measured 1 on both sides, with palpable pulsations of the posterior tibial artery. His medical history included hypertension, hyperlipidemia, heart failure, ischemic heart disease with prior coronary stent implantation, and left ventricular resection following aneurysm repair. Notably, there was no history of immunosuppression.

**Figure 3:**
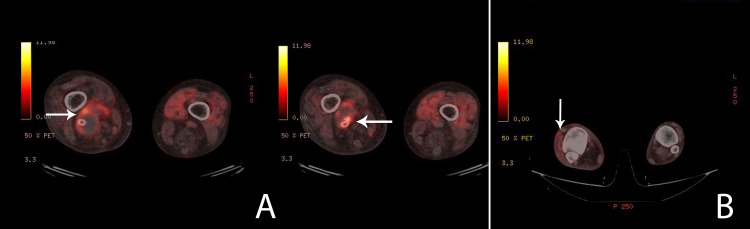
PET/CT showing increased metabolic activity. A: Increased metabolic activity in the aneurysm sac. B: Increased metabolic activity in the dorsum of the foot.

An urgent 18F-fluorodeoxyglucose positron emission tomography/computed tomography (18F-FDG PET/CT) scan revealed significant suspicion of an infectious process in the distal segment of the aneurysmal sac, the distal end of the popliteal stent graft, and the surrounding tissue in the right popliteal fossa (Figure 3). 

**Figure 4:**
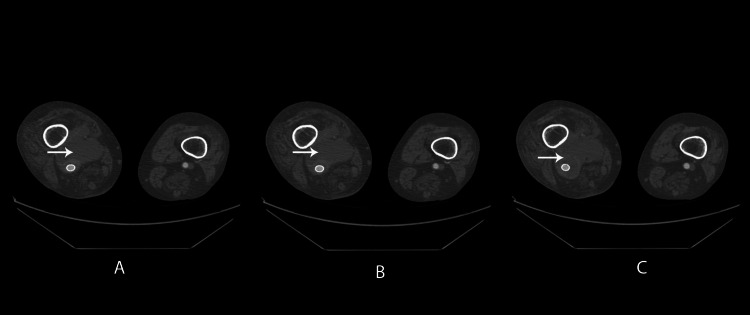
Rupture of the infected popliteal aneurysm.

Moreover, heightened activity was observed in the dorsal subcutaneous structures of the foot (Figure 3B). Great attention was focused on the abdominal region where the endovascular aneurysm repair (EVAR) had previously been performed. Fortunately, no signs of abdominal stent graft infection were identified. Subsequent contrast-enhanced CT scanning surprisingly revealed a ruptured aneurysm, despite the presence of a patent stent graft with no contrast extravasation. Notably, no gas formation was observed in the suspected area (Figure 4).

**Figure 5:**
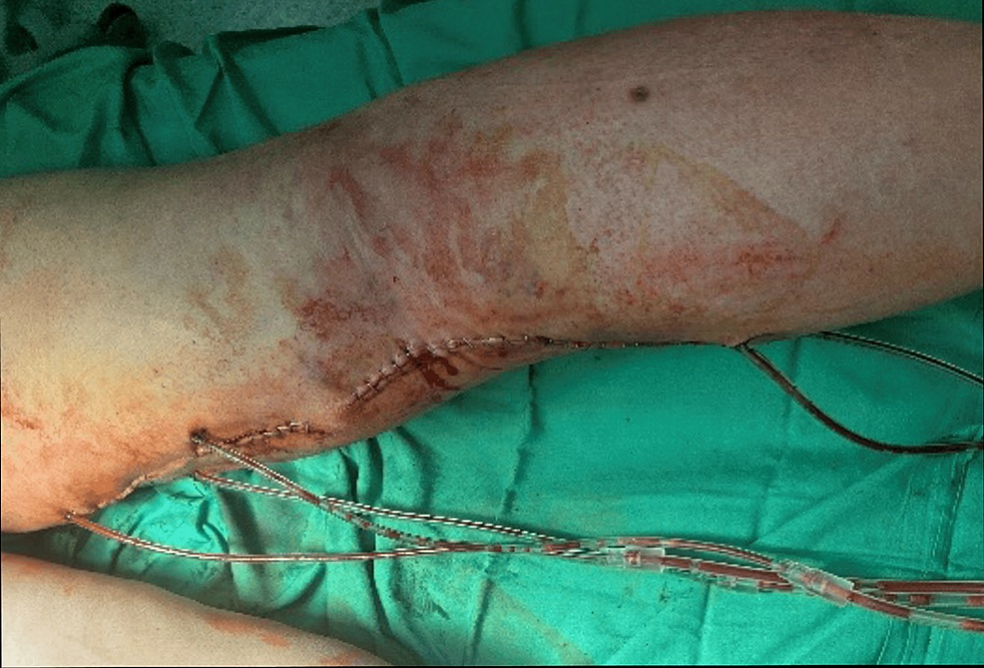
Prone position after performing the distal bypass.

A single-stage surgical procedure was conducted. Initially, the patient was positioned supine, and an extra-anatomical autovenous bypass was performed. Because of the infected operative field in the right popliteal fossa, interposition was not considered an appropriate option. The ipsilateral great saphenous vein was reversed and utilized via a medial approach. The proximal anastomosis was performed end-to-side with the proximal superficial femoral artery (about 10 cm distal to the bifurcation of the common femoral artery), while the distal anastomosis was sutured end-to-side to the tibioperoneal trunk. This position prevented undesirable angulation of the venous graft. Due to the extra-anatomical layout of the bypass, extending from a subcutaneous position below the knee to the popliteal artery, a nearly 90-degree angle of the graft would be formed. The more distal the anastomosis (e.g., the tibioperoneal trunk), the more appropriate the angle of the distal part of the graft would be. The femoral and popliteal arteries were ligated distally from the proximal anastomosis and proximally from the distal anastomosis. All surgical procedures occurred in healthy tissue without cellulitis (Figure 5).

**Figure 6:**
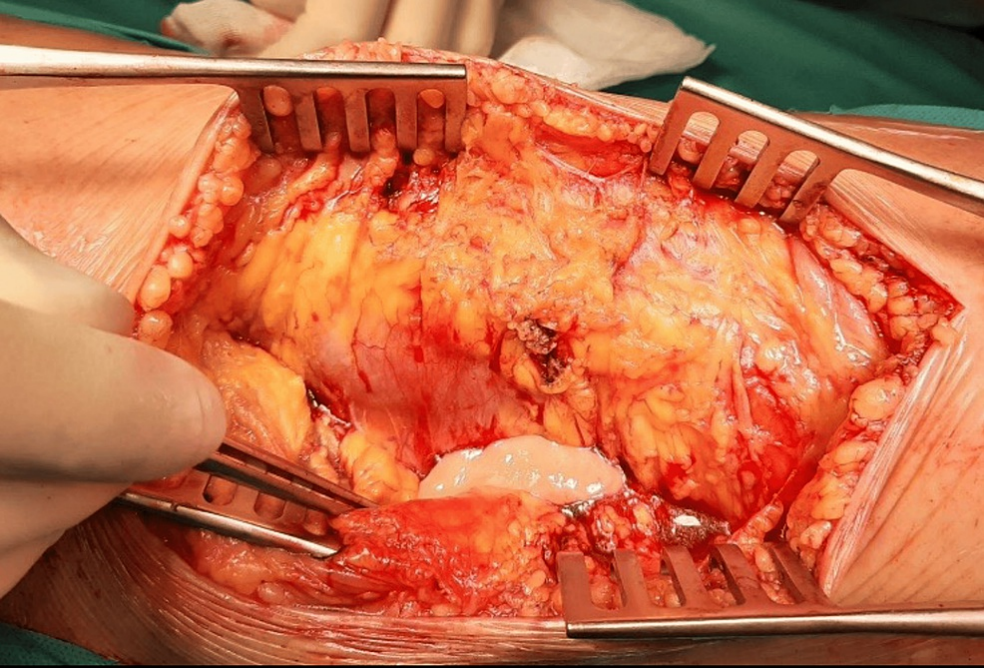
Pus in the distal segment of the aneurysm.

Post-bypass, the patient was repositioned prone. A posterior approach was used to access the popliteal fossa, revealing an aneurysm surrounded by infected tissue (Figure 6).

**Figure 7:**
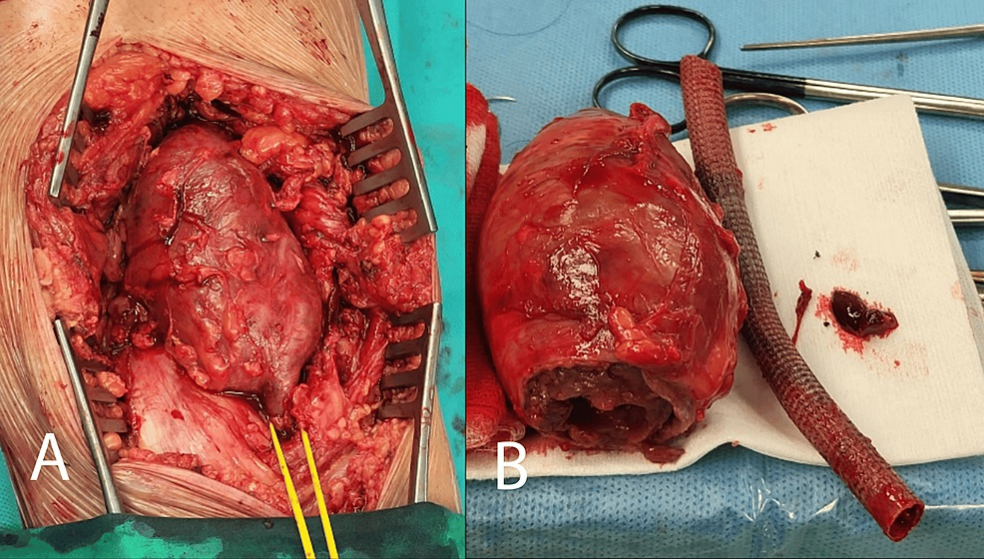
Dissected aneurysm and extracted stent graft. A: Dissected aneurysm. B: Extracted stent graft next to the explanted aneurysm.

Pus samples were collected and cultured. The aneurysm presented with a thin, fragile, edematous wall. Because of the infection, clear identification of the aneurysm's adventitia was challenging. Notably, a medial distal portion displayed an adventitial defect with infected aneurysmal thrombus propagation. The aneurysm was opened, and the thrombus was removed. The distal healthy popliteal artery was dissected and ligated post stent-graft extraction (Figure 7).

**Figure 8:**
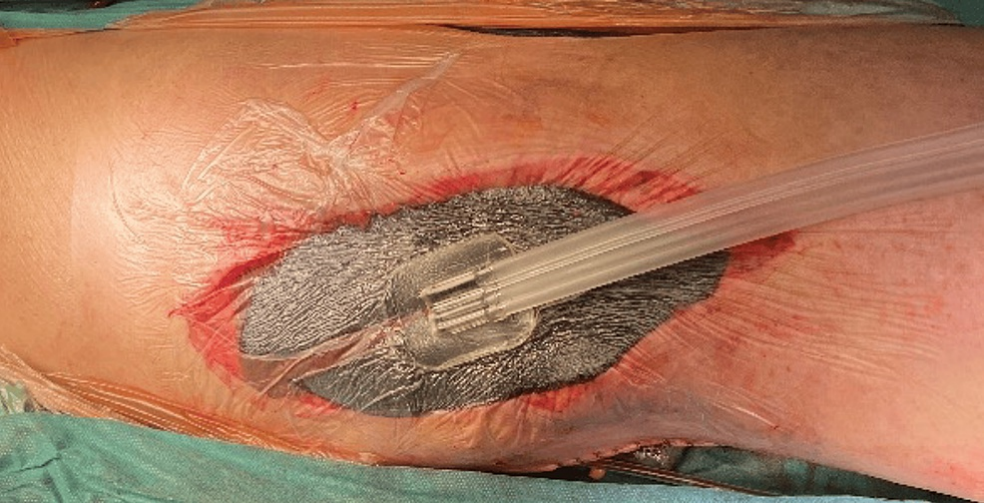
VAC system.

Proximally, extraction and artery dissection were more challenging because of aneurysmal propagation in the adductor canal. Following successful dissection, this artery segment was also ligated, and the aneurysm sac was excised. The residual cavity underwent thorough rinsing, and the wound was closed using a vacuum-assisted closure (VAC) system to conclude the procedure (Figure 8).

**Figure 9:**
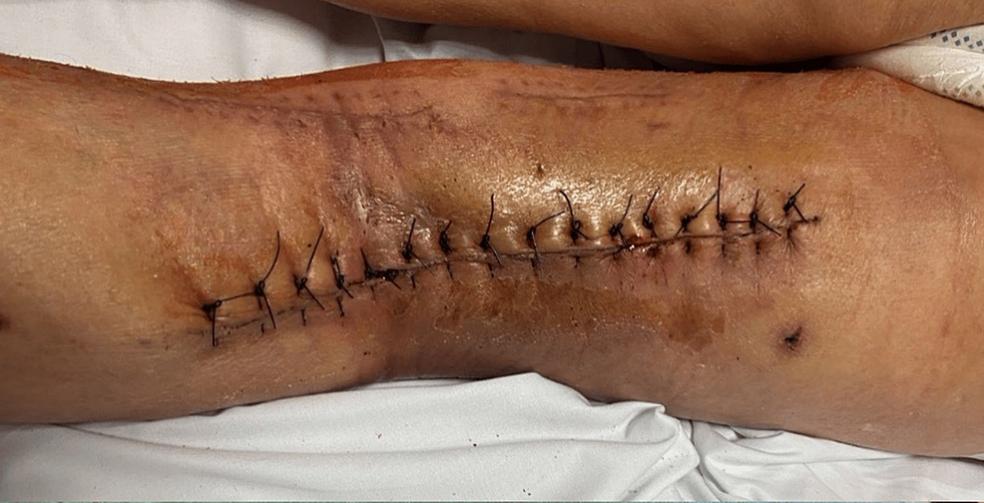
Closed popliteal access.

Cultures from the pus around the aneurysm, aneurysmal thrombus, and distal end of the stent graft tested positive for Serratia marcescens. The patient received intravenous piperacillin-tazobactam as per the antibiogram. Significant improvement in the patient's overall condition and laboratory results was observed in the early postoperative period. The popliteal wound showed minimal secretion, necessitating VAC system container changes every five to six days. Ten days post-operation, a subsequent culture from the wound revealed polyresistant Klebsiella pneumoniae alongside Serratia, prompting secondary antibiotic therapy using colistin. After four weeks, wound samples remained negative, and closure was achieved on the 37th postoperative day (Figure 9).

**Figure 10:**
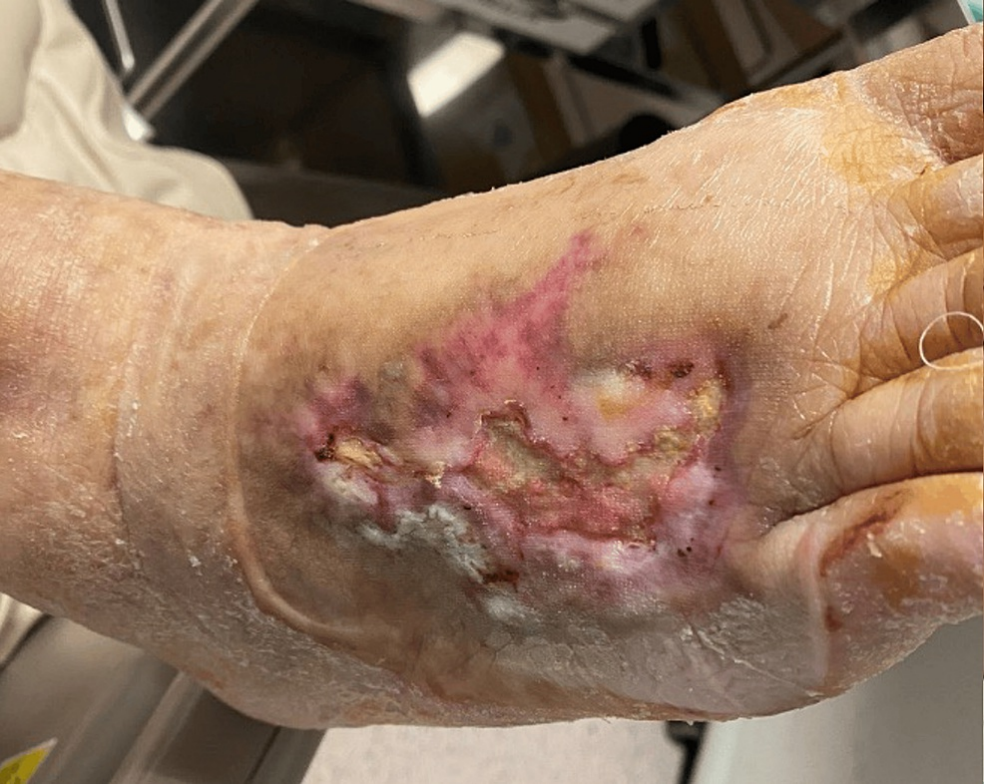
Healing process of the foot dorsum two months after surgery.

The dorsal foot lesion also exhibited notable improvement (Figure 10).

The patient was discharged 50 days after the surgery with an oral antibiotic prescription (augmentin 2 x 1,000 mg for 10 days) and clopidogrel for antiplatelet therapy. The patient was scheduled for a routine follow-up plan. Subsequent clinical follow-up indicated no signs of recurrent infection and demonstrated a patent bypass after three months.

## Discussion

Infections affecting the popliteal arteries with aneurysms are uncommon and pose both diagnostic and treatment challenges. These cases exhibit variations in anatomical location, clinical presentation, responsible pathogens, and patient age at onset [1]. Traditionally, treatment involves excision and reconstruction utilizing an autologous vein graft [2,3]. However, advancements in endovascular interventions have expanded the horizons of vascular surgery, now constituting a standard approach for aneurysmal conditions, even within the popliteal artery. In our practice, we primarily opt for the endovascular method, and, given the patient's history of myocardial infarction with coronary revascularization, followed by left ventricular aneurysm resection and ischemic stroke six months before EVAR, the endovascular approach was considered appropriate in this instance. This preference led us to initially employ a stent graft for treating the popliteal aneurysm. The Viabahn stent graft used in this case is specifically designed for this location. Recent developments have also seen the use of emergency endovascular techniques for ruptured aneurysms, proving to be valuable especially in unfit patients or those with sepsis, serving as a significant supplement to open surgery [4-6]. Nevertheless, despite the growing inclination toward endovascular repair, we advocate caution in cases of infectious aneurysms because of concerns about potential infection persistence within the stent graft. We align with the recommendation advocating the use of autologous material to prevent the risk of reinfection associated with revascularization using prosthetic grafts. Infected popliteal aneurysms are rare and have been sparsely documented in case reports [1-3]. Mycotic aneurysms develop through four mechanisms: 1) contiguous septic processes extending into periarterial lymphatic vessels and nearby arteries' vasa vasorum, 2) bacterial infection following intimal injury or atherosclerotic plaque during bacteremia, 3) direct bacterial inoculation during arterial trauma, and 4) septic embolization reaching the vasa vasorum [7]. In the case at hand, our initial observation suggested a purulent process originating in the forefoot and spreading to the popliteal area via lymphatic vessels.

Culture results from the pus around the aneurysm, thrombus, and distal end of the stent graft all indicated the same bacterial agent found in the forefoot. Consequently, we identified this as an infection stemming from the forefoot, transmitted through the lymphatic system, leading to infection of the existing popliteal aneurysm. In the post-antibiotic era, the common culprits behind mycotic aneurysms are Staphylococcus aureus, Salmonella species, and, occasionally, viridans group streptococci. Recent reports highlight the resurgence of Streptococcus pneumoniae, including penicillin-resistant strains, as a cause of mycotic aneurysms. These agents are primarily associated with septic embolization resulting from bacterial endocarditis [8,9]. Serratia marcescens, isolated in our case, is a facultative anaerobic gram-negative motile bacillus recognized for its reddish pigment production upon colonization. It can be found in soil, plants, various water sources, both natural and municipal, and at times in human intestinal flora [10]. The Serratia genus, overall, contributes to approximately 2% of nosocomial infections, spanning urinary tract infections, bloodstream infections, sepsis, pneumonia, meningoencephalitis, and other debilitating infections. However, it is rarely reported as a causative agent of mycotic aneurysms [11], primarily occurring in immunocompromised patients [12]. Additionally, a superinfection involving multidrug-resistant Klebsiella pneumoniae (likely nosocomial) was isolated from the popliteal wound a week after initiating appropriate antibiotic treatment for Serratia, which necessitates the introduction of a second antibiotic agent.

A mycotic or infected popliteal aneurysm typically manifests as a painful, throbbing swelling in the leg of febrile patients, often associated with a confirmed or unexpected infectious focus [13]. Laboratory tests often fail to definitively diagnose mycotic popliteal aneurysms, although a systemic inflammatory response is frequently observed. While inflammatory markers tend to be elevated, positive cultures are detected in only half of the cases [1]. The confirmation of an infected aneurysm often relies on imaging techniques such as CT scans, color duplex ultrasonography, and MRI. In our case, the PET/CT scan provided crucial information by precisely delineating the location and extent of the infectious process with a high level of reliability [14]. Additionally, contrast-enhanced CT scans play a pivotal role in planning vascular procedures, offering insights into inflow and outflow sites, identifying suitable venous conduits, and detailing the anatomical aspects of the aneurysm.

Treatment for infected popliteal aneurysms generally necessitates the excision of the aneurysm and infected tissues along with concurrent revascularization [2]. The surgical approach should be tailored based on specific criteria, such as the aneurysm's location, extent of infection, and presence or absence of collateral circulation. Furthermore, developing techniques to ensure effective infection control and revascularization while preventing further infection spread is essential. To prevent the spread of infection, we used separate operative fields to access the aneurysm and perform the bypass procedure. Successful treatments and revascularization of infectious popliteal aneurysms have been reported using a single-stage approach employing both posterior and medial methods, yielding satisfactory outcomes [1,2].

Postoperative antibiotic therapy is commonly indicated for a minimum duration of six weeks following surgical procedures [1]. In the instance described, antibiotic treatment was initiated and continued for two months following the surgery and two weeks after wound closure and the restoration of normal systemic inflammatory markers.

## Conclusions

This report delineates an unusual occurrence of an infected popliteal aneurysm. Our assessment indicates that the origin of the infection stemmed from ascending lymphangitis subsequent to minor surgery for purulent panaritium in the forefoot. Our intervention involved resecting the infected aneurysm and removing the previously deployed stent graft while reinstating leg circulation through an extra-anatomical bypass. A key focus during surgery was maintaining infection control by delineating distinct surgical fields. The absence of postoperative recurring infection symptoms corroborated the efficacy of our chosen strategy and surgical approach in this clinical case.
